# Radiotherapy-Activated Prodrug: Past, Present and Beyond

**DOI:** 10.1021/acscentsci.5c00875

**Published:** 2025-07-16

**Authors:** Changlun Wang, Zihang Zhang, Zhibo Liu

**Affiliations:** † Peking University-Tsinghua University Center for Life Sciences, Peking University, Beijing 100871, China; ‡ Beijing National Laboratory for Molecular Sciences, Radiochemistry and Radiation Chemistry Key Laboratory of Fundamental Science, Key Laboratory of Bioorganic Chemistry and Molecular Engineering of Ministry of Education, College of Chemistry and Molecular Engineering, 12465 Peking University, Beijing 100871, China; § Changping Laboratory, Beijing 102206, China; ∥ Key Laboratory of Carcinogenesis and Translational Research (Ministry of Education/Beijing), NMPA Key Laboratory for Research and Evaluation of Radiopharmaceuticals (National Medical Products Administration), Department of Nuclear Medicine, Peking University Cancer Hospital & Institute, Beijing 100142, China

## Abstract

Radiotherapy-activated prodrug (RAP) is defined as a type of prodrug that features low toxicity before activation, transforms into an active form upon ionizing radiation exposure, and exhibits targeted therapeutic effects within the irradiated area. In clinical practice, clear evidence demonstrates that the combination of radiotherapy and chemotherapy elicits a significant synergistic antitumor response, thereby enhancing patients’ overall survival rates. As a novel therapeutic modality, RAP has recently emerged as an active area of scientific research. The primary mechanism of RAP involves utilizing the reactive species from water radiolysis under ionizing radiation to trigger controlled cleavage of covalent bonds, enabling the controlled release of active drugs. In this Outlook, we summarize the advancements in the field of RAP, encompassing the types of ionizing radiation, novel chemical structures, and diverse prodrug formats. In addition, we discuss the current challenges and future directions of this promising field.

## Graphical abstract

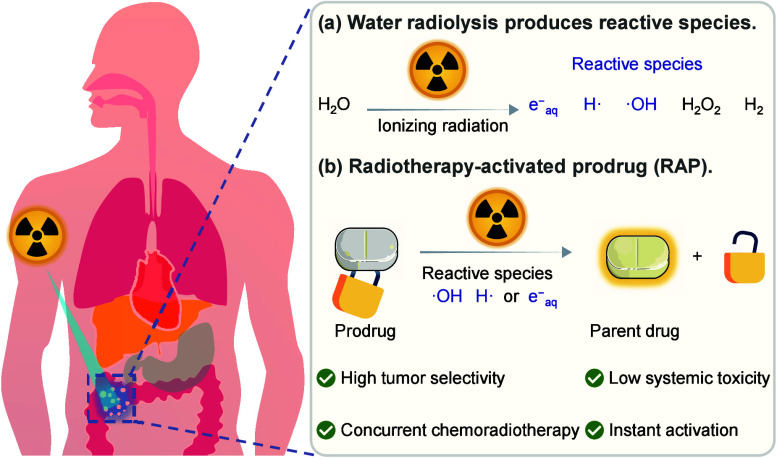

## Introduction

Cancer is a major cause of premature mortality worldwide, and it has emerged as the primary cause of mortality among individuals under the age of 70 in China, the United States and Europe.^1–3^ Chemotherapy is one of the most widely used cancer treatment modalities in clinical practice.^4^ However, systemic toxicity of chemotherapy often compromises treatment efficacy. Since the first introduction in 1958,^5^ prodrug strategies have been widely used to reduce systemic toxicity of chemotherapeutics.^6,7^ Prodrugs are derivatives of drug molecules that undergo an enzymatic and/or chemical transformation *in vivo* to release the active parent drug.^8,9^ Effectively activating the parent drugs is perhaps the most critical step of the prodrug strategy.^10^ Utilizing a clinically relevant paradigm for prodrug activation may notably reduce barriers encountered during clinical translation.

As one of the first-line cancer therapy modalities, radiotherapy benefits over 50% of cancer patients.^11^ With the development of advanced radiotherapy techniques, such as magnetic resonance imaging-guided radiotherapy and particle therapy, radiotherapy allows precise dose delivery with high spatial and temporal resolution, as well as deep tissue penetration (up to 15 cm).^12–14^ Chemoradiotherapy has been established as the standard of care for many local solid tumors, encompassing gastrointestinal malignancies, head and neck cancers, lung cancers, genitourinary cancers and so on.^15^ Clinical evidence suggests that drug-radiotherapy combinations statistically improve overall survival.^16^ Consequently, radiotherapy may act as an ideal stimulus for prodrug activation, thereby establishing an effective synergistic modality between pharmaceutical intervention and radiotherapy.

Toward radiotherapy-activated prodrug (RAP), one promising approach is to exploit reactive species during water radiolysis, which can be generated locally and confined spatially to trigger controlled bond cleavage reactions for prodrug activation. The water radiolysis process primarily generates two reactive species: hydrated electrons (e^–^_aq_) and hydroxyl radicals (•OH).^17^ Under irradiation with low-linear energy transfer (LET) radiation, such as X-ray and γ-ray, the radiolytic yields of e^–^_aq_ and •OH are both approximately 280 nM/Gy. The •OH is a highly oxidative species with a standard potential of 2.7 V, capable of inducing hydroxylation reactions of electron-rich aromatic rings and triggering oxidative processes.^18,19^ Conversely, e^–^_aq_ and hydrogen radicals (H•) are potent reducing agents, with standard potentials of −2.9 V and −2.3 V, respectively. The activation of prodrugs by radiotherapy enables the targeted release of activated chemotherapeutic agents within the tumor microenvironment. This approach effectively inhibits tumor growth while minimizing systemic side effects.

In this Outlook, we discuss the development and recent advancements of RAP, with an emphasis on reactive species and chemical reactions, and some prodrug forms of RAP are also highlighted (as shown in Figure 1). Rather than offering a comprehensive review of all notable contributions, we primarily focus on the chemical insights into the reactive species generated through water radiolysis, intending to offer inspiration for further progress in this exciting field.

**Figure 1:**
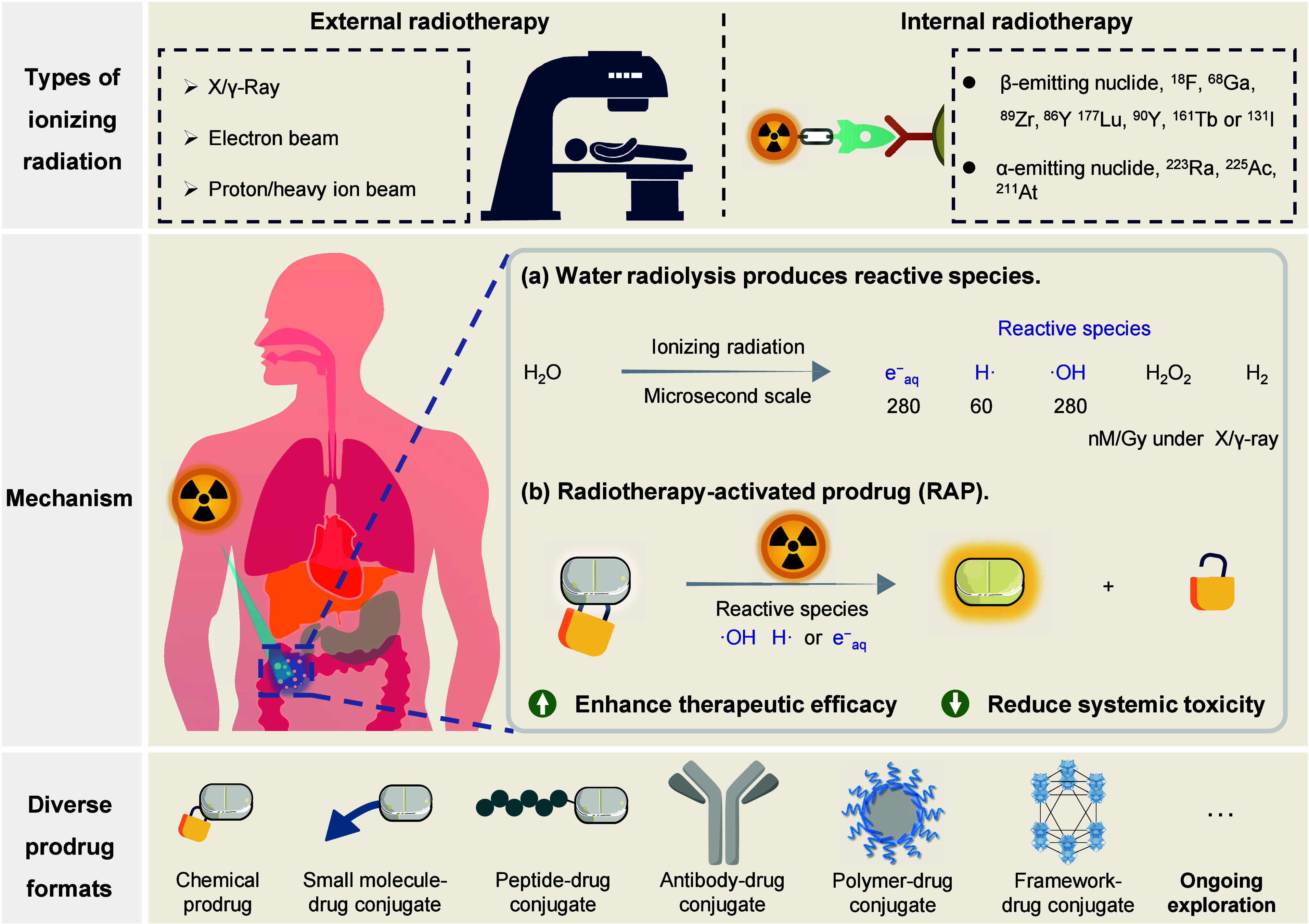
Landscape of radiotherapy-activated prodrug (RAP). Types of radiotherapy include external radiotherapy (such as photons, electrons, and charged particles) and internal radiotherapy (radionuclide). The primary mechanism of RAP is based on the reactive species generated by the radiolysis of water under ionizing radiation. The numbers beneath the reactive species correspond to their radiolytic yields. The unit “Gy” refers to the absorbed dose of ionizing radiation, which is defined as joule per kilogram of substance. Combining RAP with radiotherapy holds the potential to enhance therapeutic efficacy and reduce systemic toxicity, thus establishing a new paradigm for clinical cancer treatment. Various forms of prodrugs have been reported or are waiting to be developed.

## THE PAST OF RAP

5-Fluorouracil (5-FU), a classic antimetabolite, has been widely used in the treatment of various malignancies. 5-FU exerts its anticancer effects through the inhibition of thymidylate synthase (TS) and incorporation of its metabolites into RNA and DNA.^20,21^ In 1989, Kuroda et al. utilized γ-rays for the in situ conversion of 5-FU derivatives into 5-FU.^22^ Derivatives of 5-FU modified at the 1-position with sulfonyl and thioureido groups were efficiently converted into 5-FU (∼100 nM/Gy). Experimental results indicated that •OH and e^–^_aq_ were involved in this transformation (Table 1, entry 1, prodrug F-1, Figure 2). However, further investigations at the cellular and *in vivo* levels were not conducted in this study.

**Table 1:**
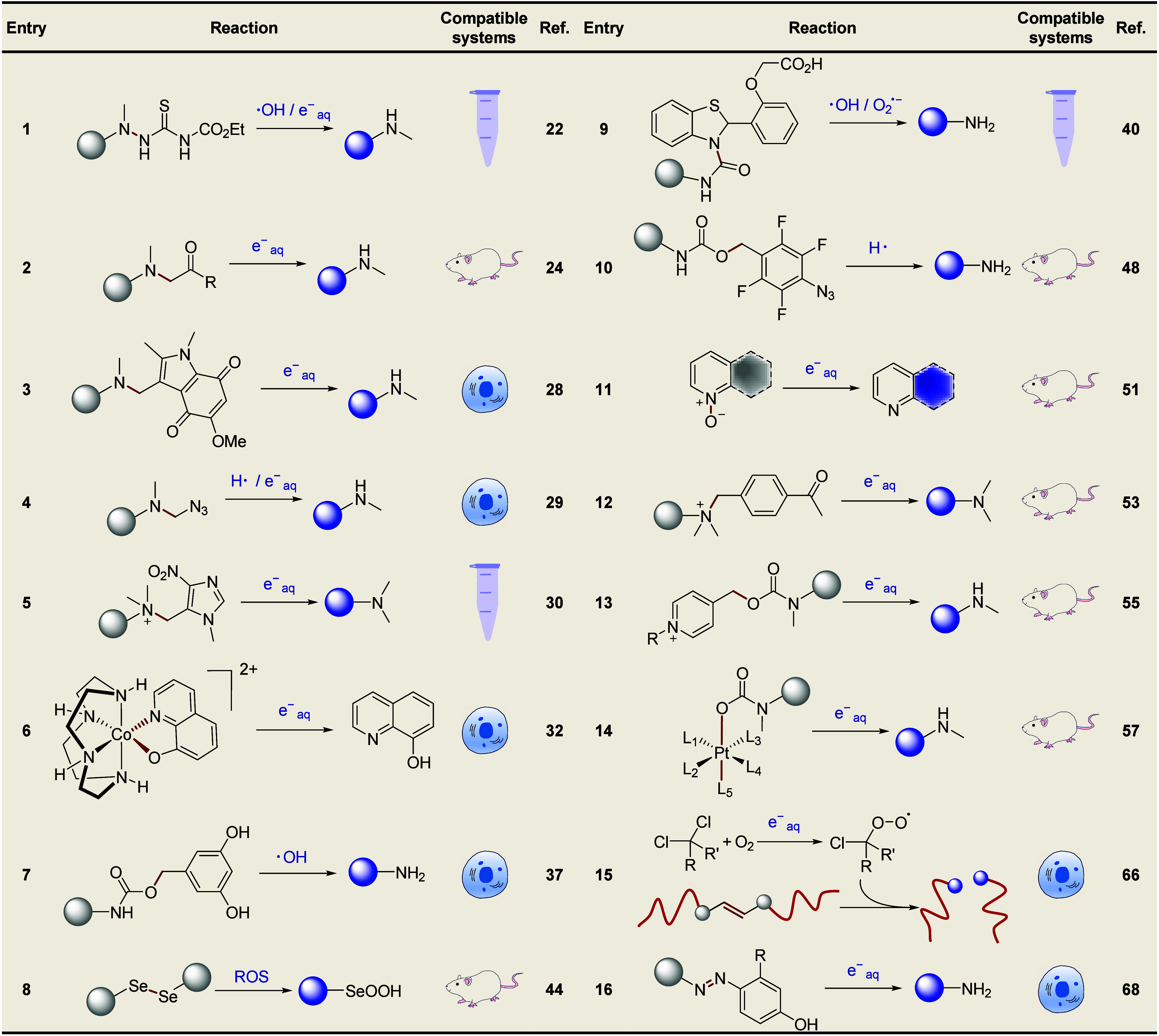
Overview of the Radiotherapy-Removable Protecting Groups (RPGs) for Prodrug Activation

**Figure 2:**
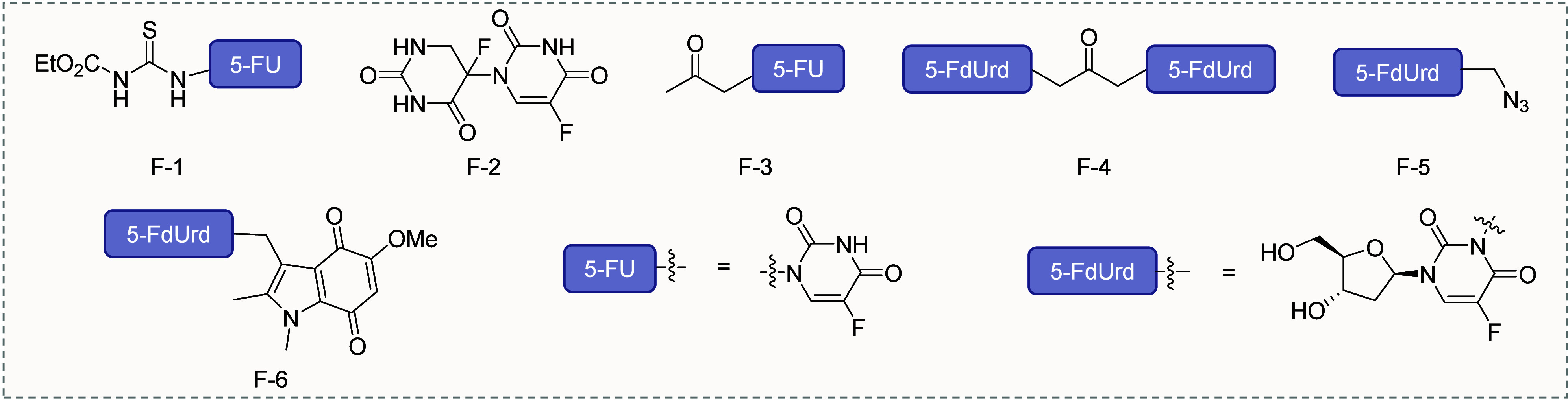
Representative RAP based on fluorouracil.

In 1992, Nishimoto et al. reported the generation of 5-FU from the N(1)–C(5′)-linked 5-FU dimer (prodrug F-2, Figure 2) in response to e^–^_aq_ (∼77 nM/Gy). The growth-delay assay of the SCCVII tumor demonstrated limited therapeutic efficacy due to the low efficiency.^23^ Furthermore, Nishimoto et al. developed a more efficient 2-oxopropyl group (Table 1, entry 2, prodrug F-3, Figure 2), with an efficiency of up to 190 nM/Gy.^24^ *In vivo* studies demonstrated that 5-FU could be released in a dose-dependent manner in the SCCVII subcutaneous tumor model, with an amount of 179 ng/g at 30 Gy, yet this is far from sufficient to provide therapeutic efficacy.^25^ To address these limitations, a more potent drug, 5-fluoro-2-deoxyuridine (FdUrd), was incorporated into the prodrug.^26,27^ By designing a 2-oxoalkyl linker, a more efficient prodrug F-4, was developed. Furthermore, Nishimoto et al. introduced azide methyl (Table 1, entry 3, prodrug F-6, Figure 2)^28^ and indolequinone (Table 1, entry 4, prodrug F-5, Figure 2)^29^ groups as radiotherapy-removable protecting groups (RPGs) for controlled drug release. However, these strategies have not yet achieved successful tumor treatment, highlighting the need for further advancements and exploration in this area.

In 1998, Wilson et al. reported the radiotherapy-sensitive heterocyclic nitroarylmethyl quaternary ammonium prodrug (Table 1, entry 5), capable of releasing chlormethine upon reduction by e^–^_aq_.^30,31^ Nevertheless, since these nitroaromatic compounds can be activated by reductases, subsequent *in vivo* investigations were not pursued. In 2004, Wilson et al. reported the utilization of Co­(III) complexes to mask the cytotoxicity of hydroxyquinoline derivatives (Table 1, entry 6), which subsequently released hydroxyquinoline toxin upon activation by e^–^_aq_.^32^ Building on these findings, they designed a Co­(III) prodrug featuring azaCBI, a potent DNA minor groove alkylator (an 8-hydroxyquinoline analog).^33^ In hypoxic human plasma, the release efficiency of azaCBI reaches 130 nM/Gy. Cellular assays showed that the azaCBI prodrug, at 1 μM, demonstrates no obvious toxicity under normoxia. However, following a 2 Gy radiation dose, the prodrug’s apparent IC_50_ in UV4 cells is significantly reduced to 0.43 nM, indicating effective activation of the prodrug under hypoxic conditions. The potent cytotoxicity of azaCBI is highly compatible with radiotherapy activation. Notably, azaCBI prodrug is susceptible to reduction under hypoxic cell culture, which may limit its application *in vivo*.

The aforementioned trials suggest that there are several challenges must be addressed before this technology can evolve into a clinical treatment paradigm: 1) Activation efficiency: Prodrugs must be efficiently activated under typical radiotherapy conditions, where 2 Gy dose per day generates around 560 nM of reactive species. This requires more effective RPGs; 2) Drug potency: The released drug must possess sufficient cytotoxic potency, ideally with an IC_50_ no higher than the nanomolar range and prolonged retention at the tumor site; 3) Prodrug stability: Given the high potency of the released drug, the prodrug must maintain stability in the physiological environment to avoid off-target toxicity until activation; 4) Optimal timing for irradiation: Pharmacokinetic studies are crucial to determine the optimal irradiation timing for efficient activation. An ideal RAP may have tumor-targeting properties, high stability, and activation mainly in response to ionizing radiation, with the released drug being potent and retaining at the tumor site. In practical scenarios, a comprehensive evaluation of various factors is essential to achieve the optimal clinical outcome. Due to these challenges and the lack of successful outcomes, the RAP field has remained dormant between 2009 and 2019. However, overcoming these hurdles could pave the way for significant advancements and clinical implementation in the future.

## The RENAISSANCE OF RAP

### RAP Triggered by Oxidative Reactive Species

Combining chemotherapy and radiotherapy has improved survival in many malignancies, which has consequently fueled ongoing investigations into the feasibility of RAP.^36^ Since 2016, as suggested by colleagues from clinical oncology, our group has been trying to develop “caging groups” that can be removed by ionizing radiation. The process is literally a failure after a failure, as we unfortunately missed most of the previous publications and there were almost no summary papers about this field. In 2020, our group reported •OH-responsive 3,5-dihydroxybenzyl carbamate (DHBC) group with an efficiency of 40 nM/Gy (Table 1, entry 7, Figure 3a).^37^ The proposed mechanism entails the electrophilic substitution of the electron-rich aromatic ring by •OH, leading to the formation of a phenol intermediate. Through spontaneous 1,4- or 1,6-elimination pathways, the intermediate can release the caged functional molecules. Monomethyl auristatin E (MMAE) is a potent mitosis inhibitor that exerts its cytotoxic effects by inhibiting tubulin polymerization, thereby inducing tumor cell death. Its IC_50_ values typically fall within the nanomolar or subnanomolar range. The DHBC-MMAE prodrug was constructed and showed no significant cytotoxicity at a concentration of 10 nM. Upon radiotherapy activation, a significant decrease in 4T1 cell viability was observed, comparable to the MMAE treatment group, thereby validating the efficacy of this RAP. However, *in vivo* experiments demonstrated a marked decrease in release efficiency, attributable to the quenching of •OH by reducing agents within the tumor’s hypoxic microenvironment.

**Figure 3:**
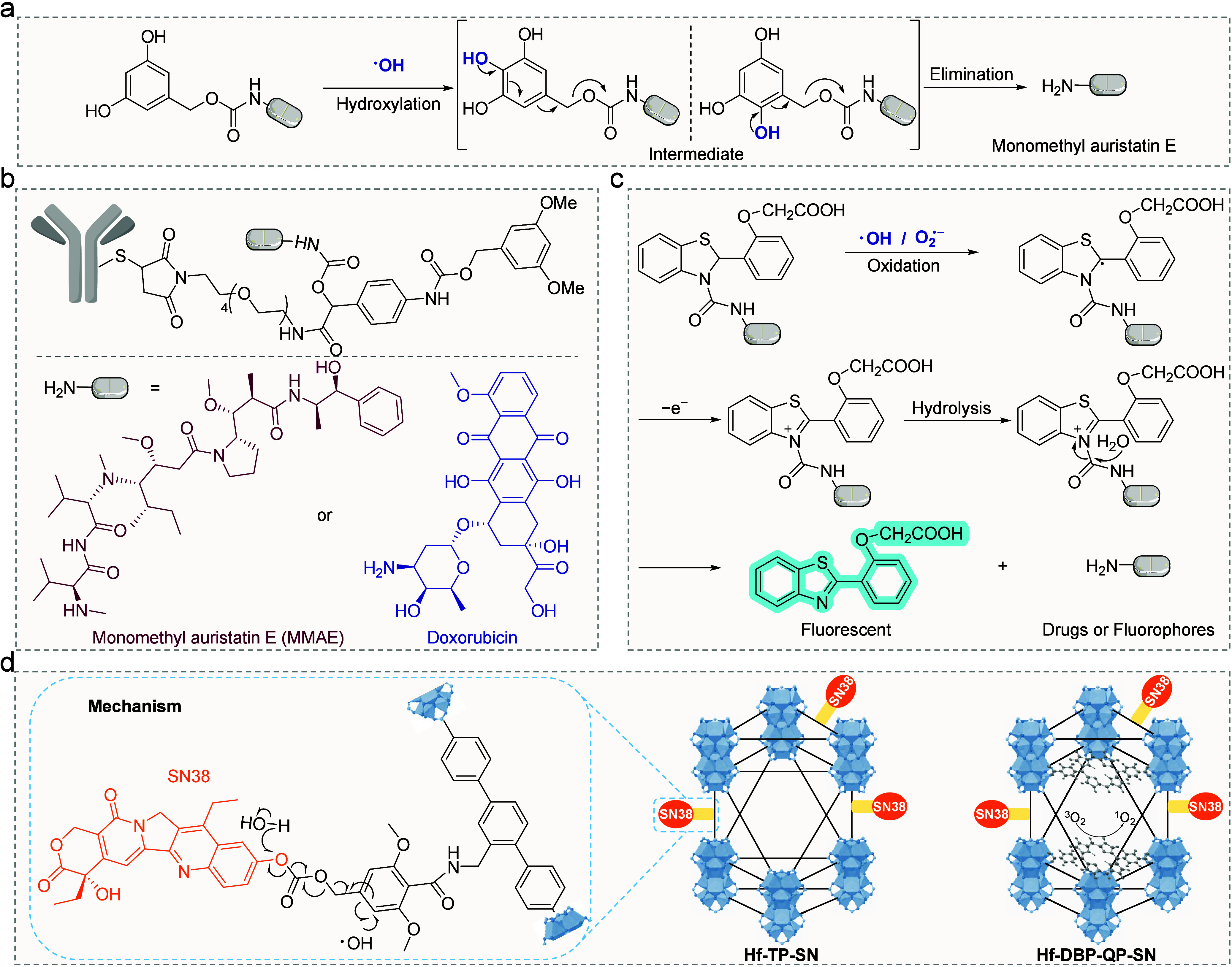
RAP-triggered by oxidative reactive species. (a) Hydroxylation of DHBC induces MMAE release. (b) Hydroxylation of DMBA induces MMAE or Dox release from ADC prodrug. (c) •OH/O_2_^•–^ activates benzothiazolines for drug release. (d) nMOF-induced enhanced local hydroxylation of DMAC for SN-38 release. Adapted with permission from refs 34 and 35. Copyright 2023 American Chemical Society and Copyright 2024 American Chemical Society, respectively.

Building upon the DHBC structure, Miller et al. constructed a targeted antibody–drug conjugate (ADC) prodrug by introducing a 3,5-dimethoxybenyl alcohol (DMBA) caging moiety in 2022 (Figure 3b).^38^ At a prodrug concentration of 10 μM, local X-ray irradiation at a clinically relevant dose of 8 Gy under hypoxic conditions facilitated the release of approximately 50% of the drug, either doxorubicin (Dox) or MMAE. In cell viability assays, the prodrugs, upon 8 Gy irradiation, demonstrated cytotoxicity similar to that of MMAE or Dox. Despite achieving promising *in vitro* results, *in vivo* experiments were not conducted due to the limited efficiency of drug release mediated by •OH.

Benzothiazolines is a classic probe used for detecting superoxide anion radicals (O_2_^•–^).^39^ In 2023, Taran et al. reported the use of benzothiazolines as cleavable linkers that release amine and carboxyl groups upon X-ray (Table 1, entry 9, Figure 3c).^40^ The proposed mechanism suggests that •OH/O_2_^•–^ abstracts a hydrogen atom, generating an unstable benzylic radical, which loses an electron to form a benzothiazolium cation. This electrophilic intermediate is then expected to undergo rapid hydrolysis, resulting in the formation of the benzothiazole molecule while simultaneously releasing the model compound. As a proof-of-concept study, benzothiazoline has been shown to respond to X-ray, releasing fluorescent molecules. However, given the complexity of the *in vivo* environment, further investigation is necessary to evaluate its potential for application in biological systems.

To successfully apply •OH-mediated controlled release reactions *in vivo*, Lin et al. developed a metal–organic framework (MOF)-based RAP.^34^ Heavy-metal-based nanoscale MOFs (nMOFs) are excellent radioenhancers by increasing energy deposition and ROS generation.^41,42^ They proposed that heavy metal nMOFs, covalently conjugated with prodrugs, could be efficiently activated by X-rays, triggering drug release through locally enhanced ROS generation (Figure 3d). By introducing the 3,5-dimethoxylbenzyl carbonate (DMBC) structure, the SN-38 prodrug was covalently conjugated to Hf-nMOF. Compared to the small molecule prodrug, the release efficiency of the nMOF prodrug under 10 Gy irradiation was five times higher (∼135 nM/Gy). Subcutaneous CT26 and 4T1 tumor models were established to assess the *in vivo* anticancer efficacy. The tumor growth curve results indicate that this RAP demonstrates impressive *in vivo* therapeutic efficacy, likely due to the synergistic effect of nMOF-mediated radiosensitization and X-ray-triggered SN-38 release. Furthermore, by incorporating bis­(*p*-benzoato)­porphyrin (DBP) ligands into the MOF, Lin constructed Hf-DBP-QP-SN.^35^ Under X-ray irradiation, not only is the toxic SN-38 released, but singlet oxygen is also generated, further enhancing the therapeutic efficacy.

Chalcogens (S, Se, and Te) have been investigated as redox-active agents in cancer therapy owing to their increased radiosensitivity.^43^ Specifically, diselenide bonds can be disrupted and oxidized to form selenite, which exhibits potent cytotoxicity toward tumor cells under γ radiation exposure (Table 1, entry 8).^44–46^ In 2024, Chen et al. reported X-ray-activated homonuclear chalcogen-embedded silica nanoplatforms, where ditellurium bonds, due to the lower bond energy, exhibit the highest sensitivity to cleavage upon X-ray irradiation.^47^ While chalcogens show sensitivity to redox species, their specificity in response to X-rays needs further refinement.

### RAP Triggered by Reductive Reactive Species

In the reductive, hypoxic tumor microenvironment, •OH is easily quenched, limiting its role in controlled release reactions. However, water radiolysis also produces reductive species, such as e^–^_aq_ and H•, which are gaining attention for their potential to drive controlled release reactions.

In 2021, Bradley et al. reported the reduction of sulfonyl azide and aryl azide to yield the corresponding amine compounds (Table 1, entry 10, Figure 4a–d).^48^ Mechanistically, they proposed that the transformation is primarily mediated by H•. Interestingly, for certain specific structures, the conversion efficiency was approximately 300 nM/Gy, whereas under X-ray irradiation, the radiolytic yield of H• is approximately 60 nM/Gy. This observation suggests that e^–^_aq_ may also contribute to the reduction process. Subsequently, a prodrug based on Dox was designed. The release efficiency of the Dox was approximately 80 nM/Gy. The 10 μM Dox prodrug showed no significant cytotoxicity, whereas, after 6 Gy irradiation, cell viability was reduced to around 50%. Through intratumoral injection of the Dox prodrug, significant inhibition of tumor growth was observed in subcutaneous HeLa tumor-bearing mice. Importantly, evaluation of body weight changes and histological abnormalities in key organs revealed no significant toxicity of the prodrug (Figure 4e,f). In comparison, the prodrug also notably reduced the cardiotoxicity of Dox.

**Figure 4:**
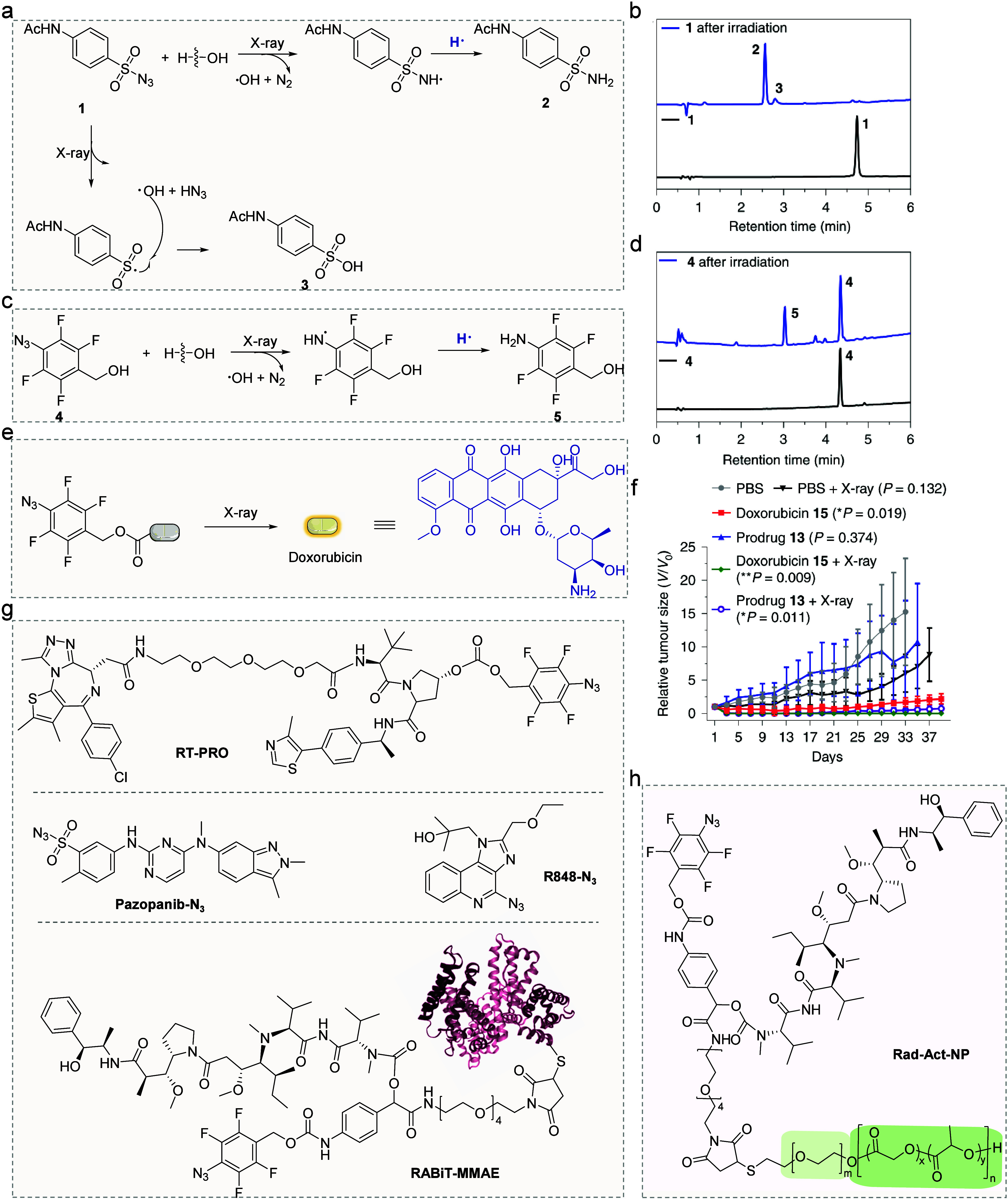
Azide-based RAPs. (a) Mechanism of the reaction of 4-acetamidobenzenesulfonyl azide **1** under X-ray irradiation. (b) HPLC traces of **1** upon irradiation (20 μM, 60 Gy) to afford compound **2** and compound **3**. (c) Mechanism of the reaction of (4-azido-2,3,5,6-tetrafluorophenyl) methanol **4** under X-ray irradiation. (d) HPLC traces of **4** upon irradiation (20 μM, 60 Gy) to afford compound **5**. (e) Azide-based Dox prodrug. (f) Subcutaneous HeLa tumor growth curve of different treatment groups. Adapted with permission from ref 48. Copyright 2021 Springer Nature. (g, h) Various azide-based RAPs.

Based on the reduction of (4-azido-2,3,5,6-tetrafluorophenyl) methanol, Li et al. reported the first example of radiotherapy-activated proteolysis-targeting chimera (PROTACs) prodrug in 2022 (Figure 4g, RT-PRO).^49^ PROTACs induce protein degradation through the ubiquitin–proteasome system (UPS) by bringing the E3 ligase and the target protein into proximity, facilitating ubiquitination and subsequent proteasomal degradation. In cell experiments, PROTAC effectively induced BRD4 degradation with a half-maximal degradation concentration (DC_50_) of 31.23 nM. In contrast, RT-PRO alone did not degrade BRD4, even at a concentration of 300 nM. However, when combined with X-ray radiation, significant BRD4 degradation was observed, resulting in a reduced DC_50_ of 8.44 nM. This novel RAP demonstrates synergistic antitumor efficacy when combined with radiotherapy in the MCF-7 xenograft model. In addition to regulating protein degradation, the azide reduction triggered by radiotherapy can be utilized for the activation of the resiquimod prodrug (R848-N_3_, Figure 4g).^50^ R848 is a small molecule agonist of Toll-like receptors 7 and 8, renowned for its ability to activate the immune response and alleviate immune suppression. This immune activation modulation not only facilitates the treatment of primary tumors but also exerts a significant inhibition on the growth of metastatic tumors. The utilization of radiotherapy to activate immune agonist prodrugs presents a promising strategy, thereby advancing the further development of RAP.

Given that daily ∼2 Gy fractions are most widely used clinically, Miller et al. developed a long-circulating prodrug, wherein MMAE or Dox prodrugs are conjugated to albumin, antibodies, or nanoparticles via a phenyl azide linker, enabling tumor targeting and extended circulation of the prodrug *in vivo*.^52^ Albumin-conjugated MMAE prodrugs exhibited a 7.5-fold higher release efficiency under the same irradiation dose compared to nonalbumin-conjugated prodrugs, significantly inhibiting tumor growth in mice (Figure 4g,h, RABiT-MMAE and Rad-Act-NP).

In 2022, our group reported two e^–^_aq_-removable protecting groups for RAP, including *N*-oxide^51^ (Table 1, entry 11) and quaternary ammonium salts (Table 1, entry 12).^53^ Among the 321 small molecule drugs approved by the FDA from 2013 to 2023, 82% contain at least one nitrogen heterocycle.^54^ Through simple one-step oxidation, nitrogen heterocyclic drugs can be converted into *N*-oxide prodrugs, where the introduction of a single oxygen atom significantly alters the physicochemical properties of the drugs, enhancing their solubility in water (Figure 5a). The proposed mechanism, depicted in Figure 5b, begins with the *N*-oxide capturing e^–^_aq_ generated by water radiolysis, forming an *N*-oxide anion radical intermediate. This intermediate then abstracts a proton from the surrounding environment and undergoes deoxidation through electron transfer, releasing a tertiary amine and a •OH. The •OH subsequently captures another e^–^_aq_, resulting in the formation of a water molecule. Furthermore, a variety of *N*-oxide prodrugs featuring π-conjugated systems can be activated by X-ray irradiation, yielding active drug release with an efficiency of approximately 140 nM/Gy (Figure 5c,d). NO-CPT has been employed for *in vivo* studies, and to determine the optimal irradiation timing, pharmacokinetics have been thoroughly investigated (Figure 5e). The optimal irradiation timing was determined to be 1 h, and this strategy successfully inhibited tumor growth.

**Figure 5:**
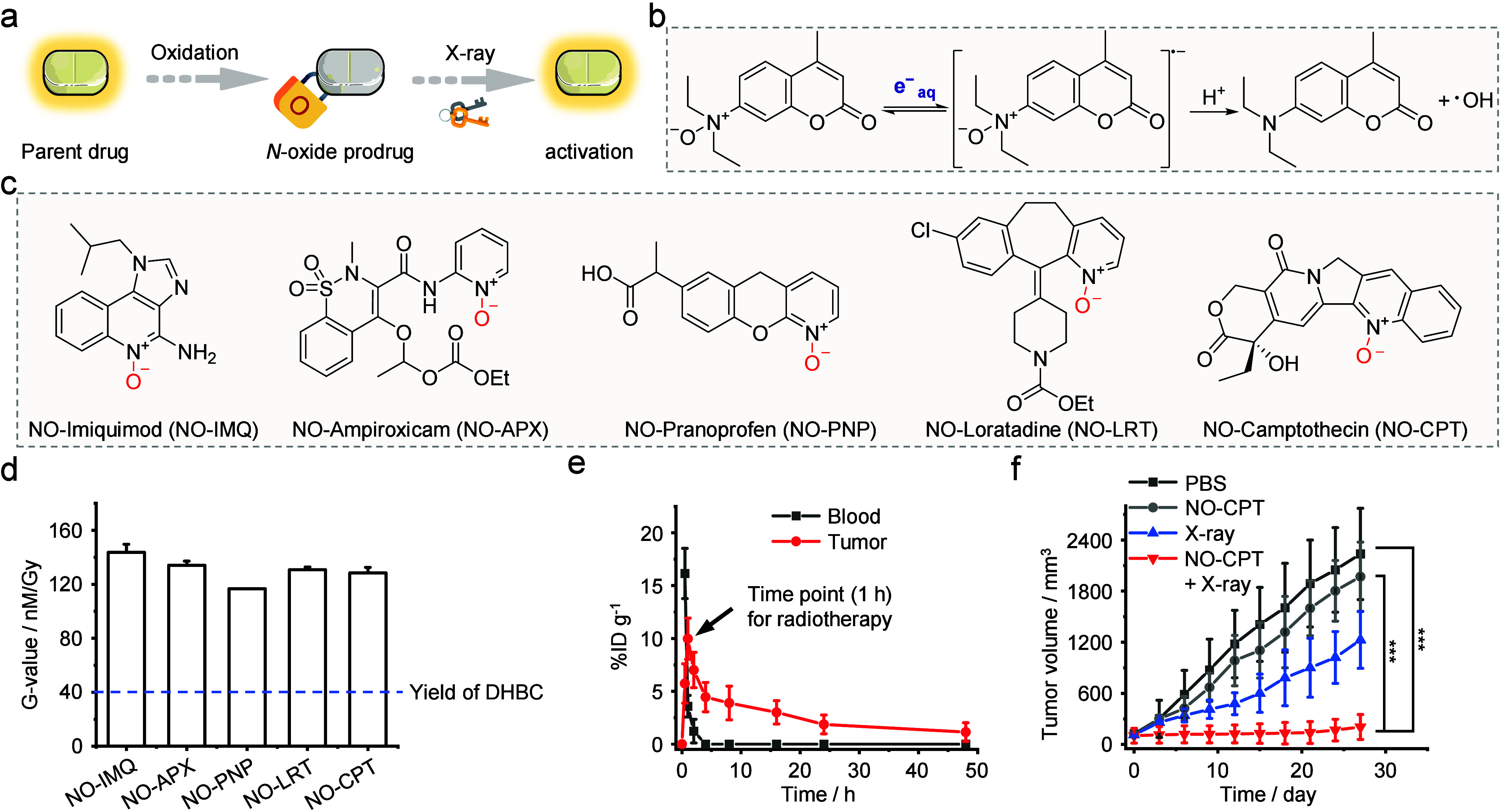
*N*-oxide-based RAPs. (a) Schematic diagram of the construction and activation of *N*-oxide prodrugs. (b) Mechanism of *N*-oxide activation by e^–^_aq_. (c) Structures of various *N*-oxide prodrugs. (d) Release efficiency of *N*-oxide prodrugs. (e) *In vivo* pharmacokinetic study of NO-CPT. (f) Tumor growth curves of HCT116 tumor-bearing mice after the indicated treatments. Adapted with permission from ref 51. Copyright 2022 American Chemical Society.

Through extensive structural screening and DFT calculations, our group developed the *p*-acetyl benzyl-quaternary ammonium (acetyl-QA, abbreviated QA) group for the controlled release of tertiary amine compounds. Various QA-based fluorophore precursors can efficiently generate fluorophores under X-ray irradiation, resulting in an enhancement of fluorescence intensity in a dose-dependent manner (Figure 6). Subsequently, the FDA-approved anticancer drug carfilzomib was designed as a QA-carfilzomib prodrug. The IC_50_ of carfilzomib against the CT26 cell line is 0.2 μM, and *in vivo* experimental results revealed that the tertiary amine drug, carfilzomib, can be released, thereby inhibiting tumor growth. Meanwhile, various QA-based tertiary amine prodrugs can respond to X-ray irradiation, leading to their efficient release with a radiolytic yield of approximately 140 nM/Gy.

**Figure 6:**
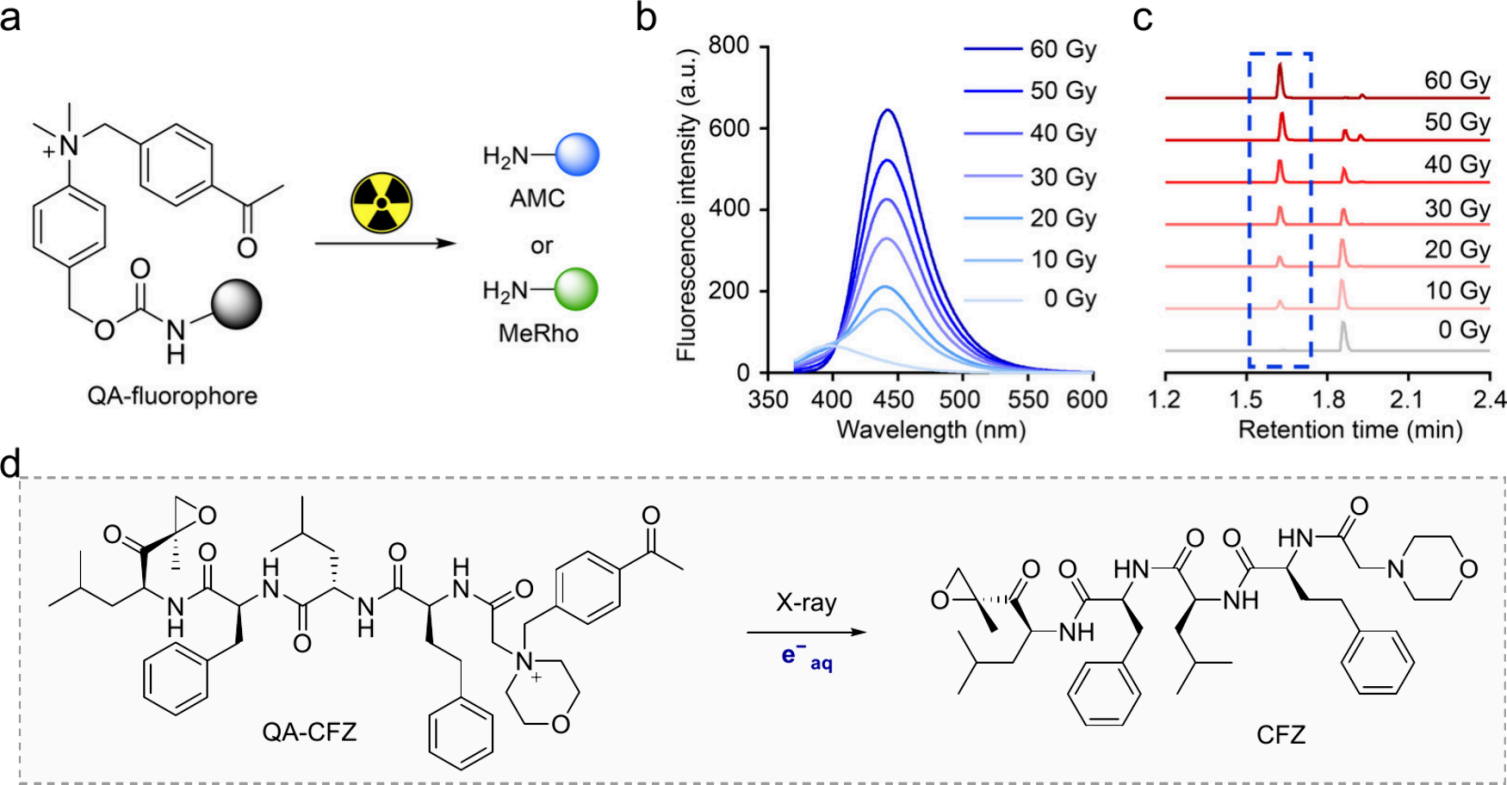
Quaternary ammonium-based RAPs. (a) Radiotherapy activates QA-fluorophore to release fluorophores. (b) The fluorescence emission spectra and (c) Ultraperformance liquid chromatography (UPLC)-UV of QA-AMC after a dose gradient of radiation (0–60 Gy). (d) e^–^_aq_ activates QA-CFZ to release CFZ. Reprinted with permission from ref 53. Copyright 2022 Wiley-VCH.

In 2024, inspired by photoinduced electron transfer chemistry, our group reported *N*-alkyl-4-pyridine (NAP) as an e^–^_aq_-removable protecting group (Table 1, entry 13).^55,56^ The proposed mechanism suggests that the aromatic ring of picolinium captures the e^–^_aq_, resulting in the formation of a neutral radical. This is followed by electron rearrangement and heterolytic cleavage of the C–O bond, which leads to the release of the client molecule and the formation of a positively charged benzyl radical. The release efficiency of different RPGs varies with experimental conditions, such as prodrug concentration, solution composition, and others. To more accurately assess the reaction efficiency of different RPGs, a head-to-head comparative assay was designed. Two radiotherapy-responsive precursors, dissolved at the same concentration in the same solution, were irradiated, and the reaction efficiency was quantified using UPLC, as shown in Figure 7a. Compared to previously reported RPGs, NAP exhibits higher reaction efficiency, facilitating the capture of e^–^_aq_ to release client molecules in complex physiological environments (Figure 7b). Furthermore, the NAP linker was developed to design an X-ray-responsive ADC (NAPC-ADC). By studying the pharmacokinetics of the ADC *in vivo*, the optimal irradiation timing was determined. In the 4T1-FAP subcutaneous tumor-bearing mice, after treatment with 4 Gy, MMAE was efficiently released from the ADC at a concentration of approximately 120 nM, leading to significant inhibition of tumor growth (Figure 7c–f).

**Figure 7:**
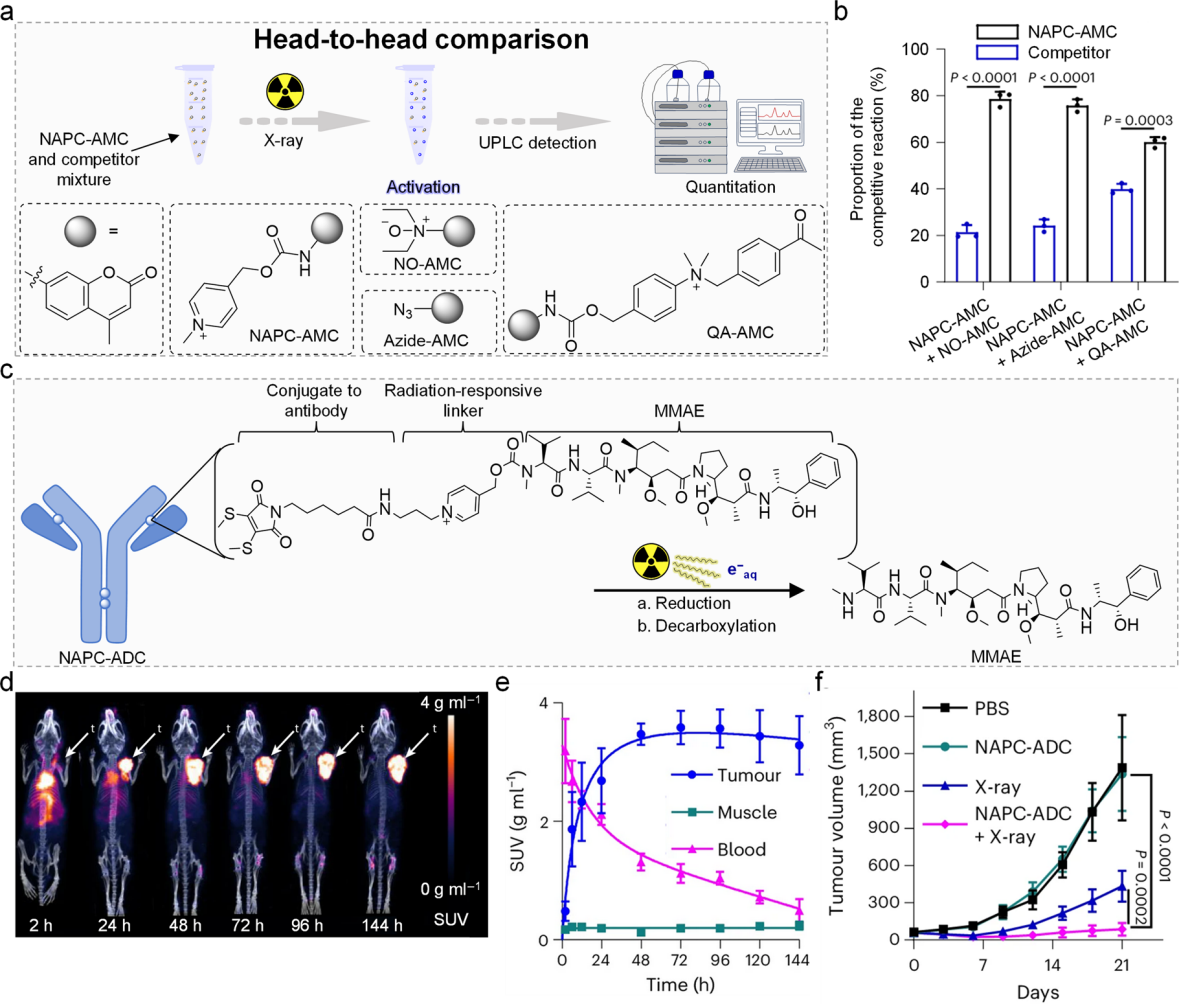
NAP-based RAPs. (a) Schematic representation of the head-to-head comparison. (b) Analysis of the head-to-head comparison between NAP and other reported RPGs. (c) Design of the NAPC-ADC conjugate. NAP linker was reduced to release MMAE. (d) Positron emission tomography-computed tomography (PET-CT) images of [^89^Zr]­Zr-NAPC-ADC in 4T1-FAP tumor-bearing mice at different time points. (e) Time activity curve of [^89^Zr]­Zr-NAPC-ADC in the blood, muscle and tumor. (f) Tumor growth curves of 4T1-FAP tumor-bearing mice after the indicated treatments. Adapted with permission from ref 55. Copyright 2024 Springer Nature.

Since cisplatin was approved as an anticancer agent in 1978, platinum-based chemotherapeutic agents remain among the most widely used anticancer drugs.^58^ Due to the severe toxicity of Pt­(II) drugs, Pt­(IV) prodrugs are considered the next generation of platinum-based drugs, with axial ligands offering novel therapeutic opportunities.^59,60^ The controlled activation of Pt­(IV) prodrugs typically relies on electron transfer-mediated reduction.^61–64^ In 2024, our group reported a radiotherapy-activated Pt­(IV) prodrug, proposing a mechanism involving the reduction of Pt­(IV) prodrug mediated by e^–^_aq_ (Table 1, entry 14).^57^ This study employs nuclear magnetic resonance (NMR) spectroscopy to characterize the release of axial ligands from Pt­(IV) prodrugs and the subsequent generation of Pt­(II) drugs (Figure 8a–c). Subsequently, an oxaliplatin-based Pt­(IV) prodrug with an acetyl group as the axial ligand was designed (OxaliPt­(IV)-(OAc)_2_). Upon X-ray irradiation, oxaliplatin was released in a dose-dependent manner, with a release efficiency of approximately 120 nM/Gy. The pharmacokinetics of OxaliPt­(IV)-(OAc)_2_ were investigated using inductively coupled plasma-mass spectrometry (ICP-MS). The tumor uptake of OxaliPt­(IV)-(OAc)_2_ peaked at 2 h post-injection and then gradually declined. In oxaliplatin-sensitive HCT116 subcutaneous tumor-bearing mice, OxaliPt­(IV)-(OAc)_2_ was efficiently activated, leading to the release of oxaliplatin and consequently significantly inhibiting tumor growth (Figure 8d–g). The identification of suitable indications is paramount to the successful application of RAP. Of note, radiotherapy-induced reduction of transition metal ions or transition metal complex is generally applicable, this provides new insights for the development of metal-based prodrugs (Figure 8h).^65^

**Figure 8:**
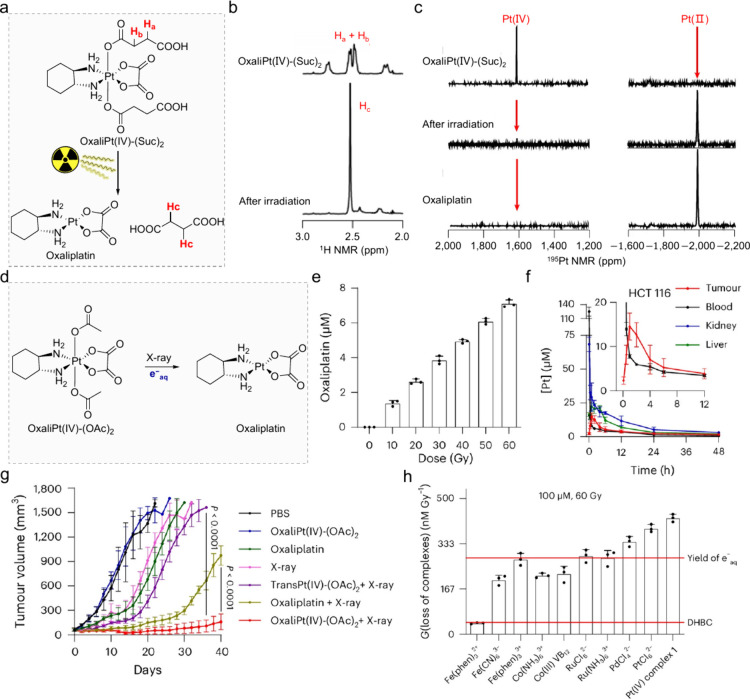
Platnium­(IV)-based RAPs. (a) OxaliPt­(IV)-(Suc)_2_ is readily reduced, releasing the Pt­(II) drug and the axial ligands. (b) ^1^H NMR and (c) ^195^Pt NMR spectra demonstrated that the axial ligands were efficiently released, simultaneously generating Pt­(II) drugs. (d) Schematic representation of X-ray-induced release of oxaliplatin. (e) Oxaliplatin released from oxaliPt­(IV)-(OAc)_2_ under clinically relevant radiation dose. (f) Pharmacokinetic studies of oxaliPt­(IV)-(OAc)_2_ in HCT116 tumor-bearing mice. (g) Tumor growth curves of HCT116 tumor-bearing mice after the indicated treatments. (h) A variety of transition metal complexes can be readily reduced by e^–^_aq_. Adapted with permission from ref 57. Copyright 2024 Springer Nature.

In addition to the aforementioned, other interesting chemical transformations driven by e^–^_aq_ have also been reported. In 2024, Eelkema et al. reported that e^–^_aq_ mediates a dechlorination reaction of organochlorides, which subsequently react with oxygen to generate highly oxidative peroxyl radicals. This process enables the controlled cleavage of the double bond motif in stilbene polymers (Table 1, entry 15).^66^ Similar peroxide species can also facilitate the oxidation of thioethers.^67^ In 2024, Ogawa et al. reported the azo-based e^–^_aq_-removable protecting group. They proposed that the cleavage mechanism involves a two-electron reduction step, which is subsequently followed by N–N bond scission, with intracellular activation further confirmed in cellular assays (Table 1, entry 16).^68^

### RAP Triggered by Internal Radiotherapy

External radiotherapy is a localized treatment modality, whereas 90% of cancer-associated mortality is attributed to metastasis.^69^ Radiopharmaceuticals, which usually are substrates or ligands labeled with radionuclides, hold increasing promise for diagnosing and treating cancer metastases.^70–72^ When radionuclides decay to emit high-energy particles (e.g., α or β particles), they may induce instant and localized water radiolysis, generating reactive species such as e^–^_aq_.^73,74^ In 2024, our group reported for the first time the use of radiopharmaceuticals for prodrug activation.^75^ 2-[^18^F]-fluoro-2-deoxy-d-glucose ([^18^F]­FDG), which decays to produce high-energy positrons, is one of the most widely used radiopharmaceuticals for cancer diagnosis and prognosis evaluation.^76^ Owing to the Warburg effect, [^18^F]­FDG exhibits selective accumulation in various tumors and is honored as the “molecule of the century”.^77,78^ The study revealed that [^18^F]­FDG can activate Pt­(IV) complexes, triggering the simultaneous release of Pt­(II) drugs and the axial ligands of original Pt­(IV) complexes in tumor. The activation may be mediated by e^–^_aq_, generated through water radiolysis resulting from the decay of the radionuclide ^18^F. When incubated with [^18^F]­FDG, OxaliPt­(IV)-Coumarin readily releases coumarin, and the yield of coumarin is dependent on the activity concentration of [^18^F]­FDG. A range of Pt­(IV) complexes can be effectively activated by [^18^F]­FDG, resulting in the release of clinically approved agents such as cisplatin, carboplatin, and oxaliplatin (Figure 9a–c). An OxaliPt­(IV) linker was developed to create an [^18^F]­FDG-activated ADC (Pt-ADC). Biodistribution studies revealed that [^18^F]­FDG and Pt-ADC exhibit tumor-specific colocalization, facilitating accurate prodrug activation through this dual-targeting approach. Additionally, this work demonstrated that previously reported RPGs can be efficiently activated by [^18^F]­FDG (Figure 9d,e). Subsequently, in addition to the radionuclide ^18^F, our group also demonstrated that several other radionuclides are capable of triggering the controllable reduction of Pt­(IV) complexes (Figure 9f).^79^ The energy deposition (Gy) contributed by radionuclides in Figure 9g is governed by three key parameters: (1) radionuclide identity (particle type [β^–^/β^+^], decay energy [keV], and half-life [t_1_/_2_]), (2) activity concentration (mCi/mL), and (3) cumulative irradiation time. For these β-emitting radionuclides, under identical activity concentrations and specified time points, prolonged half-lives and higher particle energies synergistically enhance cumulative energy deposition. By analyzing the half-life and decay energy of various radionuclides with an activity concentration of 1 mCi/mL, we established a correlation between the absorbed radiation dose and cumulative irradiation time using Geant4 simulation software. The results showed that the release of axial ligands was dependent on the absorbed radiation dose, with a linear relationship observed, emphasizing the potential for designing on-demand controlled release systems based on precise radiation dose predictions. Given its superior release efficiency and therapeutic potential, Lutetium-177 (^177^Lu) was selected for further testing. An OxaliPt­(IV)-Gem prodrug was designed with gemcitabine as the axial ligand. ^177^Lu was delivered to the tumor via FAP-targeting antibodies, followed by the injection of the prodrug OxaliPt­(IV)-Gem. *In vivo* experiments on 4T1-FAP tumor-bearing mice demonstrated robust antitumor efficacy. More importantly, this radionuclide activation strategy was effective against metastatic tumors, significantly reducing pulmonary nodules (Figure 9h,i). Meanwhile, Miller et al. demonstrated that a range of β-emitting radionuclides, as well as the α-emitting radionuclide Radium-223, are capable of triggering azide reduction reactions.^80^ The observed reduction in prodrug activation efficiency exhibited by ^223^Ra compared to β-emitters under an equivalent absorbed dose may be attributable to its characteristic high linear energy transfer (LET).^81^ Subsequently, *in vivo* prodrug activation was achieved through long-circulating albumin-based prodrugs and targeted β-emitting radiopharmaceuticals. However, the therapeutic efficacy of internal radiotherapy-activated prodrugs *in vivo* requires further investigation, as α-emitting radionuclides exhibit higher relative biological effectiveness (RBE) than β-emitting radionuclides under an equivalent absorbed dose, necessitating more comprehensive studies to delineate α-emitting and β-emitting radionuclides combined therapeutic outcomes with prodrug activation systems.

**Figure 9:**
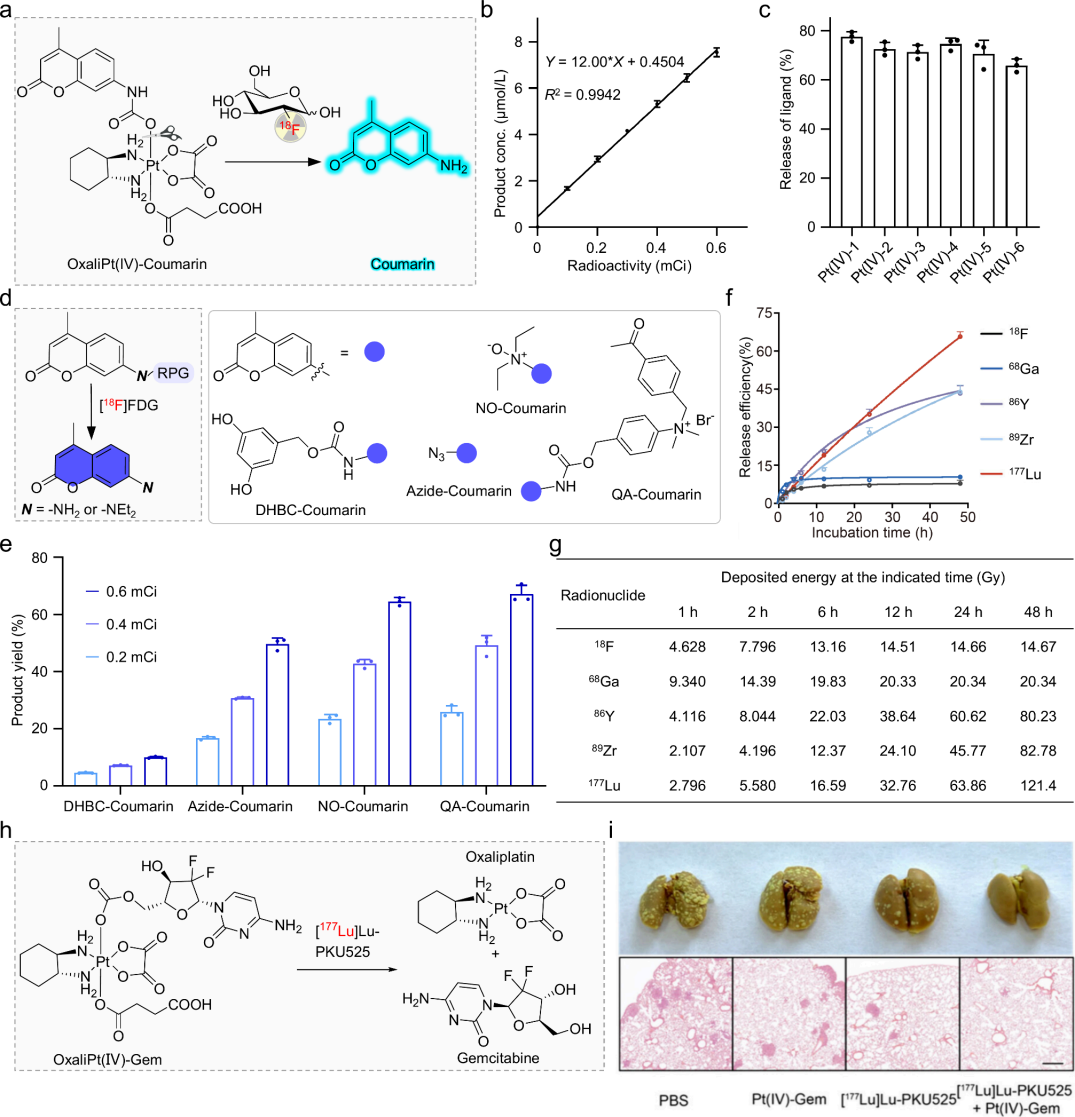
Radionuclide-triggered prodrug activation. (a) [^18^F]­FDG triggers the release of coumarin from OxaliPt­(IV)-Coumarin. (b) The yield of coumarin exhibits a linear relationship with the activity concentration of [^18^F]­FDG. (c) [^18^F]­FDG-triggered reduction is generally applicable for a range of Pt­(IV) complexes. (d, e) [^18^F]­FDG activates previously reported RPGs. Adapted with permission from ref 75. Copyright 2024 Elsevier. (f) Various radionuclides trigger the release of axial ligands from Pt­(IV) complexes. (g) Energy deposit at the indicated time of tested radionuclides simulated by Geant4. (h) ^177^Lu triggers the release of oxaliplatin and gemcitabine from the OxaliPt­(IV)-Gem prodrug. (i) ^177^Lu activates OxaliPt­(IV)-Gem prodrug in the 4T1-FAP lung metastasis tumor model significantly reduced pulmonary nodules. Adapted with permission from ref 79. Copyright 2024 American Chemical Society.

## CONCLUSIONS AND OUTLOOK

RAP presents a promising strategy to mitigate chemotherapy-induced systemic toxicity and enables concurrent chemoradiotherapy, offering the potential to enhance therapeutic efficacy while minimizing side effects, thereby establishing a novel paradigm in clinical cancer treatment. This Outlook highlights the activation of prodrugs through reactive species generated by water radiolysis and summarizes the latest advancements in the field. Compared to X-ray-activated nanosystems,^82–87^ RAP remains in its infancy. However, the potential advantages of RAP, including enhanced tumor permeability, scalable batch production capability, and more established detection and analytical methodologies in pharmacokinetic research, have attracted growing attention from researchers in recent years. On the other hand, compared to enzyme-activated prodrugs relying on radiotherapy-induced upregulated enzymes,^88,89^ RAP may exhibit potential advantages, such as higher tumor selectivity and lower tumor heterogeneity. As an emerging field, RAP still has several key directions yet to be explored. More specifically, these directions primarily involve the following four aspects:

### Types of Ionizing Radiation

Due to the Bragg peak, charged particle therapy is considered one of the most advanced radiation therapy techniques.^90^ Owing to its higher relative biological effectiveness, heavy ion radiotherapy (e.g., carbon ion therapy) has garnered increasing attention. Currently, prodrug activation driven by carbon ion beams has not yet been explored. Furthermore, as heavy ions may offer superior therapeutic effects, the combination with immune agonist prodrugs could potentially yield unexpected therapeutic outcomes in the future. Recent advancements in the field of radiopharmaceuticals have opened new opportunities for the development of RAP.^91^ Covalently targeted radioligands significantly prolong the retention of radionuclides at the tumor site, which is crucial for radionuclide-activated RAP. The coexistence time of the radionuclide and prodrug at the tumor site directly determines the activation efficiency of the prodrug.

### Chemistry of RAP

RPGs determine the efficiency of drug release. In the development of novel RPGs, head-to-head comparison should be incorporated to ensure that the new RPGs possess enhanced competitive binding affinity for e^–^_aq_ in complex physiological environments. The integration of artificial intelligence (AI) with quantum mechanical simulations accelerates the rational design of RPGs. By computing critical parameters, (e.g., vertical electron affinities and bond dissociation activation energies), these tools elucidate radical-mediated cleavage mechanisms and establish mechanistic frameworks for developing diverse RPGs. In addition, new reaction modalities are crucial, such as radiotherapy-driven controllable bond-formation reactions for in situ drug molecule synthesis, which are also worth exploring. Theoretically, in situ synthesis of drug molecules could create a wider therapeutic window between the prodrug and the active drug, thereby further reducing the toxicity of the prodrug.^92–94^ On the other hand, novel reaction mechanisms are equally intriguing.^95^ In summary, there is a strong demand for the exploration of new chemical tools to accommodate a wide range of future biomedical applications.

### Diverse Prodrug Formats

A variety of prodrug formats have been widely explored, including small molecule prodrugs, nanoparticle-based prodrugs, ADCs, and albumin-conjugated prodrugs.^37,38,51–53,55,57^ Nevertheless, there remain many prodrug formats that are still worth exploring. Cao et al. proposed an intriguing strategy of using e^–^_aq_ to reduce Pd ions for the construction of Pd(0) catalysts. Yet, the *in vivo* applicability of this approach remains to be further investigated.^96^ Additionally, the regulation of enzyme function holds significant potential due to the well-established catalytic efficiency and specificity of enzymes.^97,98^ Constructing a radiotherapy-responsive proenzyme could dramatically enhance catalytic activity upon irradiation, thus improving the efficiency of drug release and broadening the therapeutic applications of radiotherapy in drug delivery systems.^99–101^

### The Indications for RAP

As a novel therapeutic modality, RAP has the potential to address a broad spectrum of indications. Initially, priority should be given to cancers where chemoradiotherapy is already an established standard treatment. In these cases, the introduction of RAPs can enhance therapeutic efficacy without significantly altering the current regimen, making them a seamless complement to existing therapies. Unlike traditional concurrent chemoradiotherapy, RAP holds the potential to significantly minimize systemic toxicity that often limits the long-term use of chemotherapy.^102,103^ Furthermore, tumors exhibiting dual sensitivity to both chemotherapy and radiotherapy represent the optimal candidates for RAP, as RAP can synergize with radiotherapy to maximize antitumor effects while minimizing toxicity. Additionally, neoadjuvant chemoradiotherapy is considered the clinical standard of care for locally advanced cancers, such as breast cancer and rectal cancer.^104,105^ RAP is anticipated to become the standard regimen for neoadjuvant therapy. In the future, RAPs are anticipated to be utilized for indications associated with resistance to radiotherapy or chemotherapy. RAP presents innovative strategies for cancer therapy, paving the way for the clinical application of radiotherapy-induced precision chemotherapy with enhanced efficacy and minimal side effects. We have reasons to believe that RAP may be launched for clinical studies in the near future.

## References

[1] Sung H., Ferlay J., Siegel R. L., Laversanne M., Soerjomataram I., Jemal A., Bray F. (2021). Global cancer statistics
2020: Globocan estimates of incidence and mortality worldwide for
36 cancers in 185 countries. CA Cancer J. Clin..

[2] Bray F., Laversanne M., Sung H., Ferlay J., Siegel R. L., Soerjomataram I., Jemal A. (2024). Global cancer statistics 2022: Globocan
estimates of incidence and mortality worldwide for 36 cancers in 185
countries. CA. Cancer J. Clin..

[3] Han B., Zheng R., Zeng H., Wang S., Sun K., Chen R., Li L., Wei W., He J. (2024). Cancer incidence
and mortality in China, 2022. J. Natl. Cancer.
Cent..

[4] Advancing cancer therapy [Editorial]. Nat. Cancer 2021, 2, 245–246 10.1038/s43018-021-00192-x. 35121963

[5] Albert A. (1958). Chemical aspects
of selective toxicity. Nature.

[6] Fu Q., Shen S., Sun P., Gu Z., Bai Y., Wang X., Liu Z. (2023). Bioorthogonal chemistry
for prodrug
activation *in vivo*. Chem. Soc.
Rev..

[7] Ji X., Pan Z., Yu B., De la Cruz L., Zheng Y., Ke B., Wang B. (2019). Click and release: bioorthogonal approaches to ’’on-demand’’
activation of prodrugs. Chem. Soc. Rev..

[8] Rautio J., Kumpulainen H., Heimbach T., Oliyai R., Oh D., Jarvinen T., Savolainen J. (2008). Prodrugs: design and clinical applications. Nat. Rev. Drug Discovery.

[9] Fralish Z., Chen A., Khan S., Zhou P., Reker D. (2024). The landscape
of small-molecule prodrugs. Nat. Rev. Drug Discovery.

[10] Cheetham A., Chakroun R., Ma W., Cui H. (2017). Self-assembling prodrugs. Chem. Soc. Rev..

[11] Petroni G., Cantley L., Santambrogio L., Formenti S., Galluzzi L. (2022). Radiotherapy
as a tool to elicit clinically actionable signalling pathways in cancer. Nat. Rev. Clin. Oncol..

[12] Lan G., Ni K., Xu R., Lu K., Lin Z., Chan C., Lin W. (2017). Nanoscale metal-organic
layers for deeply penetrating X-ray-induced
photodynamic therapy. Angew. Chem., Int. Ed..

[13] Loeffler J., Durante M. (2013). Charged particle therapy-optimization, challenges and
future directions. Nat. Rev. Clin. Oncol..

[14] Keall P., Brighi C., Glide-Hurst C., Liney G., Liu P., Lydiard S., Paganelli C., Pham T., Shan S., Tree A., van der Heide U., Waddington D., Whelan B. (2022). Integrated MRI-guided radiotherapy-opportunities and
challenges. Nat. Rev. Clin. Oncol..

[15] Farrer N., Higgins G., Kunkler I. (2022). Radiation-induced
prodrug activation:
extending combined modality therapy for some solid tumours. Br. J. Cancer.

[16] Sharma R., Plummer R., Stock J., Greenhalgh T., Ataman O., Kelly S., Clay R., Adams R., Baird R., Billingham L., Brown S., Buckland S., Bulbeck H., Chalmers A., Clack G., Cranston A., Damstrup L., Ferraldeschi R., Forster M., Golec J., Hagan R., Hall E., Hanauske A., Harrington K., Haswell T., Hawkins M., Illidge T., Jones H., Kennedy A., McDonald F., Melcher T., O’Connor J., Pollard J., Saunders M., Sebag-Montefiore D., Smitt M., Staffurth J., Stratford I., Wedge S. (2016). Clinical development of new drug-radiotherapy
combinations. Nat. Rev. Clin. Oncol..

[17] Le
Caër S. (2011). Water radiolysis: influence of oxide surfaces on H_2_ production under ionizing radiation. Water.

[18] Li H., Li X., Shi W., Xu Y., Ma H. (2018). Rationally designed
fluorescence •OH probe with high sensitivity and selectivity
for monitoring the generation of •OH in iron autoxidation without
addition of H_2_O_2_. Angew.
Chem., Int. Ed..

[19] Berndt T., Richters S., Jokinen T., Hyttinen N., Kurtén T., Otkjær R. V., Kjaergaard H. G., Stratmann F., Herrmann H., Sipilä M., Kulmala M., Ehn M. (2016). Hydroxyl radical-induced
formation of highly oxidized organic compounds. Nat. Commun..

[20] Longley D., Harkin D., Johnston P. (2003). 5-Fluorouracil: Mechanisms of action
and clinical strategies. Nat. Rev. Cancer.

[21] Vodenkova S., Buchler T., Cervena K., Veskrnova V., Vodicka P., Vymetalkova V. (2020). 5-Fluorouracil
and other fluoropyrimidines
in colorectal cancer: Past, present and future. Pharmacol. Ther..

[22] Kuroda T., Hisamura K., Matsukuma I., Nishikawa H., Nakamizo N. (1989). γ-Radiolysis of 1-substituted 5-fluorouracil
derivatives. Bull. Chem. Soc. Jpn..

[23] Nishimoto S., Hatta H., Ueshima H., Kagiya T. (1992). 1-(5′-Fluoro-6’-hydroxy-5′,6’-dihydrouracil-5′-yl)-5-fluorouracil,
a novel N(1)-C(5)-linked dimer that releases 5-fluorouracil by radiation
activation under hypoxic conditions. J. Med.
Chem..

[24] Shibamoto Y., Zhou L., Hatta H., Mori M., Nishimoto S.-i. (2000). A novel
class of antitumor prodrug, 1-(2′-oxopropyl)-5-fluorouracil
(OFU001), that releases 5-fluorouracil upon hypoxic irradiation. Jpn. J. Cancer Res..

[25] Shibamoto Y., Zhou L., Hatta H., Mori M., Nishimoto S.-I. (2001). In vivo
evaluation of a novel antitumor prodrug, 1-(2′-oxopropyl)-5-fluorouracil
(OFU001), which releases 5-fluorouracil upon hypoxic irradiation. Int. J. Radiat. Oncol. Biol. Phys..

[26] Tanabe K., Mimasu Y., Eto A., Tachi Y., Sakakibara S., Mori M., Hatta H., Nishimoto S. (2003). One-electron
reduction characteristics of N(3)-substituted 5-fluorodeoxyuridines
synthesized as radiation-activated prodrugs. Bioorg. Med. Chem..

[27] Tanabe K., Sugiura M., Ito T., Nishimoto S. i. (2012). Synthesis
and one-electron reduction characteristics of radiation-activated
prodrugs possessing two 5-fluorodeoxyuridine units. Bioorg. Med. Chem..

[28] Tanabe K., Hirata N., Harada H., Hiraoka M., Nishimoto S. I. (2008). Emission
under hypoxia: One-electron reduction and fluorescence characteristics
of an indolequinone-coumarin conjugate. ChemBioChem..

[29] Tanabe K., Ishizaki J., Ando Y., Ito T., Nishimoto S.-i. (2012). Reductive
activation of 5-fluorodeoxyuridine prodrug possessing azide methyl
group by hypoxic X-irradiation. Bioorg. Med.
Chem. Lett..

[30] Wilson W., Tercel M., Anderson R., Denny W. (1998). Radiation-activated
prodrugs as hypoxia-selective cytotoxins: model studies with nitroarylmethyl
quaternary salts. Anti-Cancer Drug Des..

[31] Kriste A., Tercel M., Anderson R., Ferry D., Wilson W. (2002). Pathways of
reductive fragmentation of heterocyclic nitroarylmethyl quaternary
ammonium prodrugs of mechlorethamine. Radiat.
Res..

[32] Ahn G., Ware D., Denny W., Wilson W. (2004). Optimization of the
auxiliary ligand shell of cobalt­(III)­(8-hydroxyquinoline) complexes
as model hypoxia-selective radiation-activated prodrugs. Radiat. Res..

[33] Ahn G., Botting K., Patterson A., Ware D., Tercel M., Wilson W. (2006). Radiolytic and cellular
reduction of a novel hypoxia-activated
cobalt­(III) prodrug of a chloromethylbenzindoline DNA minor groove
alkylator. Biochem. Pharmacol..

[34] Xu Z., Zhen W., McCleary C., Luo T., Jiang X., Peng C., Weichselbaum R., Lin W. (2023). Nanoscale metal-organic
framework with an X-ray triggerable prodrug for synergistic radiotherapy
and chemotherapy. J. Am. Chem. Soc..

[35] Zhen W., Xu Z., Mao Y., McCleary C., Jiang X., Weichselbaum R. R., Lin W. (2024). Nanoscale mixed-ligand metal-organic framework for X-ray stimulated
cancer therapy. J. Am. Chem. Soc..

[36] Lawrence Y., Vikram B., Dignam J., Chakravarti A., Machtay M., Freidlin B., Takebe N., Curran W., Bentzen S., Okunieff P., Coleman C., Dicker A. (2013). NCI-RTOG translational
program strategic guidelines for the early-stage development of radiosensitizers. J. Natl. Cancer Inst..

[37] Fu Q., Li H., Duan D., Wang C., Shen S., Ma H., Liu Z. (2020). External-radiation-induced
local hydroxylation enables remote release
of functional molecules in tumors. Angew. Chem.,
Int. Ed..

[38] Quintana J., Arboleda D., Hu H., Scott E., Luthria G., Pai S., Parangi S., Weissleder R., Miller M. (2022). Radiation cleaved drug-conjugate
linkers enable local payload release. Bioconjugate
Chem..

[39] Zhang L., Tang B., Ding Y. (2005). Study of 2-(2-pyridyl)­benzothiazoline
as a novel fluorescent probe for the identification of superoxide
anion radicals and the determination of superoxide dismutase activity
in scallion genus foods. J. Agric. Food Chem..

[40] Tuo W., Renault J., Rajpal A., Pin S., Le Gall T., Taran F. (2023). Radiation-responsive benzothiazolines
as potential cleavable fluorogenic
linkers for drug delivery. Chem.Eur. J..

[41] Lu K., He C., Guo N., Chan C., Ni K., Lan G., Tang H., Pelizzari C., Fu Y.-X., Spiotto M. T., Weichselbaum R. R., Lin W. (2018). Low-dose X-ray radiotherapy-radiodynamic
therapy via nanoscale metal-organic frameworks enhances checkpoint
blockade immunotherapy. Nat. Biomed. Eng..

[42] Ni K., Lan G., Lin W. (2020). Nanoscale metal-organic frameworks generate reactive
oxygen species for cancer therapy. ACS Cent.
Sci..

[43] Chang Y. Z., Huang J. R., Shi S. J., Xu L. G., Lin H., Chen T. F. (2023). Precise Engineering of a Se/Te Nanochaperone for Reinvigorating
Cancer Radio-Immunotherapy. Adv. Mater..

[44] Li T., Pan S., Gao S., Xiang W., Sun C., Cao W., Xu H. (2020). Diselenide-pemetrexed assemblies for combined cancer immuno-, radio-,
and chemotherapies. Angew. Chem., Int. Ed..

[45] Gao S., Li T., Guo Y., Sun C., Xianyu B., Xu H. (2020). Selenium-containing
nanoparticles combine the NK cells mediated immunotherapy with radiotherapy
and chemotherapy. Adv. Mater..

[46] Shao D., Zhang F., Chen F., Zheng X., Hu H., Yang C., Tu Z., Wang Z., Chang Z., Lu J., Li T., Zhang Y., Chen L., Leong K., Dong W. (2020). Biomimetic
diselenide-bridged mesoporous organosilica nanoparticles
as an X-ray-responsive biodegradable carrier for chemo-immunotherapy. Adv. Mater..

[47] You Y., Chang Y., Pan S., Bu Q., Ling J., He W., Chen T. (2025). Cleavage of homonuclear
chalcogen-chalcogen bonds in
a hybrid platform in response to X-ray radiation potentiates tumor
radiochemotherapy. Angew. Chem., Int. Ed..

[48] Geng J., Zhang Y., Gao Q., Neumann K., Dong H., Porter H., Potter M., Ren H., Argyle D., Bradley M. (2021). Switching on prodrugs using radiotherapy. Nat. Chem..

[49] Yang C., Yang Y., Li Y., Ni Q., Li J. (2023). Radiotherapy-triggered
proteolysis targeting chimera prodrug activation in tumors. J. Am. Chem. Soc..

[50] Sun J. L., Yao H. C., Ren X. T., Cui L. J., Liu L. L., Wang G. Q., Tang Z. H. (2024). Radiation-activated
resiquimod prodrug
nanomaterials for enhancing immune checkpoint inhibitor therapy. Nano Lett..

[51] Ding Z., Guo Z., Zheng Y., Wang Z., Fu Q., Liu Z. (2022). Radiotherapy
reduces *N*-oxides for prodrug activation in tumors. J. Am. Chem. Soc..

[52] Quintana J., Kang M., Hu H., Ng T., Wojtkiewicz G., Scott E., Parangi S., Schuemann J., Weissleder R., Miller M. (2024). Extended pharmacokinetics improve
site-specific prodrug activation using radiation. ACS Cent. Sci..

[53] Guo Z., Hong H., Zheng Y., Wang Z., Ding Z., Fu Q., Liu Z. (2022). Radiotherapy-induced
cleavage of quaternary ammonium
groups activates prodrugs in tumors. Angew.
Chem., Int. Ed..

[54] Marshall C., Federice J., Bell C., Cox P., Njardarson J. (2024). An update
on the nitrogen heterocycle compositions and properties of US FDA-approved
pharmaceuticals (2013–2023). J. Med.
Chem..

[55] Fu Q., Gu Z., Shen S., Bai Y., Wang X., Xu M., Sun P., Chen J., Li D., Liu Z. (2024). Radiotherapy
activates
picolinium prodrugs in tumours. Nat. Chem..

[56] Edson J., Spencer L., Boncella J. (2011). Photorelease of primary aliphatic
and aromatic amines by visible-light-induced electron transfer. Org. Lett..

[57] Fu Q., Zhang S., Shen S., Gu Z., Chen J., Song D., Sun P., Wang C., Guo Z., Xiao Y., Gao Y., Guo Z., Liu Z. (2024). Radiotherapy-triggered
reduction of platinum-based chemotherapeutic prodrugs in tumours. Nat. Biomed. Eng..

[58] Rottenberg S., Disler C., Perego P. (2021). The rediscovery
of platinum-based
cancer therapy. Nat. Rev. Cancer.

[59] Hall M., Mellor H., Callaghan R., Hambley T. (2007). Basis for design and
development of platinum­(IV) anticancer complexes. J. Med. Chem..

[60] Johnstone T., Suntharalingam K., Lippard S. (2016). The next generation of platinum drugs:
targeted Pt­(II) agents, nanoparticle delivery, and Pt­(IV) prodrugs. Chem. Rev..

[61] Wang Z., Wang N., Cheng S., Xu K., Deng Z., Chen S., Xu Z., Xie K., Tse M., Shi P., Hirao H., Ko C., Zhu G. (2019). Phorbiplatin,
a highly
potent Pt­(IV) antitumor prodrug that can be controllably activated
by red light. Chem..

[62] Deng Z., Wang N., Liu Y., Xu Z., Wang Z., Lau T., Zhu G. (2020). A photocaged, water-oxidizing,
and nucleolus-targeted
Pt­(IV) complex with a distinct anticancer mechanism. J. Am. Chem. Soc..

[63] Liang G., Sadhukhan T., Banerjee S., Tang D., Zhang H., Cui M., Montesdeoca N., Karges J., Xiao H. (2023). Reduction of platinum­(IV)
prodrug hemoglobin nanoparticles with deeply penetrating ultrasound
radiation for tumor-targeted therapeutically enhanced anticancer therapy. Angew. Chem., Int. Ed..

[64] Liu G., Zhang Y., Yao H., Deng Z., Chen S., Wang Y., Peng W., Sun G., Tse M., Chen X., Yue J., Peng Y., Wang L., Zhu G. (2023). An ultrasound-activatable platinum
prodrug for sono-sensitized chemotherapy. Sci.
Adv..

[65] Graf N., Lippard S. (2012). Redox activation of metal-based prodrugs as a strategy
for drug delivery. Adv. Drug Delivery Rev..

[66] Liu J., Brevé T., Xu B., Hagedoorn P., Denkova A., Eelkema R. (2024). Organochlorides mediate
oxidation
reactions induced by low dose ionizing radiation. CCS Chem..

[67] Xu Y., Mu B., Tu Z., Liang W., Li J., Sang Z., Liu Z. (2025). Radiation-induced
aerobic oxidation via solvent-derived peroxyl radicals. Chem. Sci..

[68] Ogawara K., Inanami O., Takakura H., Saita K., Nakajima K., Kumar S., Ieda N., Kobayashi M., Taketsugu T., Ogawa M. (2024). Theoretical design
and synthesis
of caged compounds using X-ray-triggered azo bond cleavage. Adv. Sci..

[69] Chaffer C., Weinberg R. (2011). A perspective on cancer
cell metastasis. Science.

[70] Dolgin E. (2018). Radioactive
drugs emerge from the shadows to storm the market. Nat. Biotechnol..

[71] Bodei L., Herrmann K., Schoder H., Scott A., Lewis J. (2022). Radiotheranostics
in oncology: current challenges and emerging opportunities. Nat. Rev. Clin. Oncol..

[72] Sgouros G., Bodei L., McDevitt M., Nedrow J. (2020). Radiopharmaceutical
therapy in cancer: clinical advances and challenges. Nat. Rev. Drug Discovery.

[73] Kotagiri N., Laforest R., Achilefu S. (2018). Reply to ‘Is
Cherenkov luminescence
bright enough for photodynamic therapy?’. Nat. Nanotechnol..

[74] Fukumura T., Nakao R., Yamaguchi M., Suzuki K. (2004). Stability of ^11^C-labeled PET radiopharmaceuticals. Appl. Radiat.
Isot..

[75] Wang C., Xu M., Zhang Z., Zeng S., Shen S., Ding Z., Chen J., Cui X.-Y., Liu Z. (2024). Locally unlocks prodrugs
by radiopharmaceutical in tumor for cancer therapy. Sci. Bull..

[76] Almuhaideb A., Papathanasiou N., Bomanji J. (2011). [^18^F]­FDG PET/CT imaging
in oncology. Ann. Saudi Med..

[77] Tekade R., Sung X. (2017). The Warburg effect
and glucose-derived cancer theranostics. Drug
Discovery Today.

[78] Lan L., Liu H., Wang Y., Deng J., Peng D., Feng Y., Wang L., Chen Y., Qiu L. (2022). The potential utility
of [^68^Ga]­Ga-DOTA-FAPI-04 as a novel broad-spectrum oncological
and non-oncological imaging agent-comparison with [^18^F]­FDG. Eur. J. Nucl. Med. Mol. Imaging.

[79] Guo Z., Wang X., Han Y., Shen S., Tian P., Hu Y., Ding Z., Fu Q., Liu Z. (2024). Targeted radionuclide
therapy activates prodrugs for treating metastasis. ACS Cent. Sci..

[80] Quintana J., Jiang F., Kang M., Onecha V., Könik A., Qin L., Rodriguez V., Hu H., Borges N., Khurana I., Banla L., Le Fur M., Caravan P., Schuemann J., Bertolet A., Weissleder R., Miller M., Ng T. (2025). Localized
in vivo prodrug activation using radionuclides. J. Nucl. Med..

[81] Baba K., Kusumoto T., Okada S., Ogawara R., Kodaira S., Raffy Q., Barillon R., Ludwig N., Galindo C., Peaupardin P., Ishikawa M. (2021). Quantitative estimation
of track
segment yields of water radiolysis species under heavy ions around
Bragg peak energies using Geant4-DNA. Sci. Rep..

[82] Kang M., Quintana J., Hu H., Teixeira V., Olberg S., Banla L., Rodriguez V., Hwang W., Schuemann J., Parangi S., Weissleder R., Miller M. (2024). Sustained and localized
drug depot release using radiation-activated scintillating nanoparticles. Adv. Mater..

[83] Liu H., Zhao J., Xue Y., Zhang J., Bai H., Pan S., Peng B., Li L., Voelcker N. (2023). X-ray-induced drug
release for cancer therapy. Angew. Chem., Int.
Ed..

[84] Ruan Y., Huang H., Liu H., Gong J., Li C., Sun X. (2025). X-ray controlled release strategy in cancer therapy. BMEMat.

[85] Chen X., Song J., Chen X., Yang H. (2019). X-ray-activated nanosystems
for theranostic applications. Chem. Soc. Rev..

[86] Chen F., Ruan F., Xie X., Lu J., Sun W., Shao D., Chen M. (2024). Gold nanocluster: A
photoelectric
converter for X-ray-activated chemotherapy. Adv. Mater..

[87] Liao Y., Wang D., Gu C., Wang X., Zhu S., Zheng Z., Zhang F., Yan J., Gu Z. (2024). A cuproptosis
nanocapsule for cancer radiotherapy. Nat. Nanotechnol..

[88] Cho Y., Chung S., Kim H., Won T., Choi J., Kim I., Kim S., Byun Y. (2019). The novel
strategy for concurrent
chemoradiotherapy by conjugating the apoptotic cell-binding moiety
to caspase-3 activated doxorubicin prodrug. J. Controlled Release.

[89] Chung S., Cho Y., Choi J., Kim H., Won T., Kim S., Byun Y. (2019). Highly potent monomethyl auristatin
E prodrug activated by caspase-3
for the chemoradiotherapy of triple-negative breast cancer. Biomaterials.

[90] Durante M., Debus J., Loeffler J. (2021). Physics and
biomedical challenges
of cancer therapy with accelerated heavy ions. Nat. Rev. Phys..

[91] Cui X.-Y., Li Z., Kong Z., Liu Y., Meng H., Wen Z., Wang C., Chen J., Xu M., Li Y., Gao J., Zhu W., Hao Z., Huo L., Liu S., Yang Z., Liu Z. (2024). Covalent targeted radioligands
potentiate
radionuclide therapy. Nature.

[92] Clavadetscher J., Hoffmann S., Lilienkampf A., Mackay L., Yusop R., Rider S., Mullins J., Bradley M. (2016). Copper catalysis in
living systems and in situ drug synthesis. Angew.
Chem., Int. Ed..

[93] Clavadetscher J., Indrigo E., Chankeshwara S., Lilienkampf A., Bradley M. (2017). In-cell dual drug synthesis by cancer-targeting
palladium
catalysts. Angew. Chem., Int. Ed..

[94] Maslah H., Skarbek C., Gourson C., Plamont M., Pethe S., Jullien L., Le Saux T., Labruère R. (2021). In-cell generation
of anticancer phenanthridine through bioorthogonal cyclization in
antitumor prodrug development. Angew. Chem.,
Int. Ed..

[95] Huang J., Su L., Xu C., Ge X., Zhang R., Song J., Pu K. (2023). Molecular radio afterglow probes for cancer radiodynamic theranostics. Nat. Mater..

[96] Cao W., Wang J., Liang Y., Wu S., Deng R., Zhu H. (2024). Radiotherapy mediated catalytic prodrug
therapy with higher radiochemical
conversion than hydrated electrons. ChemRxiv.

[97] Hou J., Pan Y., Zhu D., Fan Y., Feng G., Wei Y., Wang H., Qin K., Zhao T., Yang Q., Zhu Y., Che Y., Liu Y., Cheng J., Kong D., Wang P., Shen J., Zhao Q. (2019). Targeted delivery of
nitric oxide via a ’bump-and-hole’-based enzyme-prodrug
pair. Nat. Chem. Biol..

[98] Wang J., Liu Y., Liu Y., Zheng S., Wang X., Zhao J., Yang F., Zhang G., Wang C., Chen P. R. (2019). Time-resolved
protein activation by proximal decaging in living systems. Nature.

[99] Tanabe K., Ebihara M., Hirata N., Nishimoto S.-i. (2008). Radiolytic
one-electron reduction characteristics of tyrosine derivative caged
by 2-oxopropyl group. Bioorg. Med. Chem. Lett..

[100] Zheng Q., Ma T., Wang M. (2024). Unleashing the power
of proenzyme delivery for targeted therapeutic applications using
biodegradable lipid nanoparticles. Acc. Chem.
Res..

[101] Yang Y., Aloysius H., Inoyama D., Chen Y., Hu L. (2011). Enzyme-mediated hydrolytic activation of prodrugs. Acta Pharm. Sin B.

[102] Seiwert T. Y., Salama J. K., Vokes E. E. (2007). The concurrent
chemoradiation
paradigm - general principles. Nat. Clin. Pract.
Oncol..

[103] Li X., Sun H., Lu Y., Xing L. (2022). Radiotherapy-triggered
prodrug activation: A new era in precise chemotherapy. Med..

[104] Yang Y., Pang K., Lin G., Liu X., Gao J., Zhou J., Xu L., Gao Z., Wu Y., Li A., Han J., Wu G., Wang X., Li F., Ye Y., Zhang J., Chen G., Wang H., Kong Y., Wu A., Xiao Y., Yao H., Zhang Z. (2025). Neoadjuvant chemoradiation
with or without PD-1 blockade in locally advanced rectal cancer: a
randomized phase 2 trial. Nat. Med..

[105] Huang E., Tucker S., Strom E., McNeese M., Kuerer H., Hortobagyi G., Buzdar A., Valero V., Perkins G., Schechter N., Hunt K., Sahin A., Buchholz T. (2005). Predictors of locoregional
recurrence in patients with
locally advanced breast cancer treated with neoadjuvant chemotherapy,
mastectomy, and radiotherapy. Int. J. Radiat.
Oncol. Biol. Phys..

